# MLKL, a new actor of UVB-induced apoptosis in human diploid dermal fibroblasts

**DOI:** 10.1038/s41420-024-02004-4

**Published:** 2024-05-14

**Authors:** Anne-Sophie Gary, Sophie Amouret, Alicia Montoni, Patrick J. Rochette

**Affiliations:** 1 Centre de Recherche du CHU de Québec – Université Laval, Axe Médecine Régénératrice, Hôpital du Saint-Sacrement, Québec, QC Canada; 2 Centre de Recherche en Organogénèse Expérimentale de l’Université Laval/LOEX, Université Laval, Québec, QC Canada; 3 Département d’Ophtalmologie et ORL - chirurgie cervico-faciale, Université Laval, Québec, QC Canada

**Keywords:** Apoptosis, Cancer

## Abstract

Ultraviolet radiation (UVR) is a major environmental mutagen. In skin, UVR can initiate cancer through the induction of mutagenic DNA damage and promote its progression. An important cancer prevention mechanism is the regulated cell death (RCD), which can safely dispose of damaged cells. Apoptosis, a well-known RCD, is known to be activated by UVR, but part of the mechanism and proteins involved in UVR-induced apoptosis are still to be discovered. Receptor-interacting serine/threonine-protein kinase 3 (RIPK3) and mixed lineage kinase domain-like (MLKL) are two proteins involved in necroptosis, a form of RCD. Here, we have evaluated the implication of RIPK3 and MLKL in UVB-induced cell death in human diploid dermal fibroblasts. Our results show that RIPK3 and MLKL play opposite roles in UVB-induced cell death, in a necroptosis independent pathway. We showed that RIPK3 protects cells from UVB cell death, while MLKL sensitizes cells to UVB-induced apoptosis. Taken together these results are the first to show the implication of RIPK3 and MLKL in survival and apoptosis, respectively, bringing two new actors in UVB-induced cell death pathway.

## Introduction

Ultraviolet radiation (UVR), composed of UVA (315–400 nm), UVB (280–315 nm) and UVC (100 nm–280 nm), is a major environmental stress for skin. UVA and part of UVB reach the earth surface and can penetrate the skin [1–3]. They are known to cause DNA damage and cell death to skin epidermal and dermal cells [4–8], with the formation of bi-pyrimidine DNA damage and oxidative stress [9, 10]. UVB wavelengths are involved in initiation and progression of keratinocyte skin cancer [11, 12] through the formation of bi-pyrimidine DNA damage. Several mechanisms are in place to prevent the conversion of these damage into cancer-driver mutations. One mechanism is the removal of bi-pyrimidine DNA photoproducts by the nucleotide excision repair (NER) system in human cells [13, 14]. A second mechanism is the activation of regulated cell death (RCD), that can be triggered to directly eliminate damaged cells [15].

RCD is an important skin cancer prevention mechanism and regroup a plethora of pathways [16]. Among RCD, apoptosis is the most studied and is characterised by a cascade of activated caspases, leading to DNA and proteins degradations, followed by cell death [17–19]. Other RCD have been described, one of which is the necroptosis pathway. Receptor-interacting serine/threonine-protein kinase 3 (RIPK3) and mixed lineage kinase domain-like (MLKL) kinases are two main actors of this pathway [20]. RIPK3 complex formation, called necrosome, and RIPK3 phosphorylation induce the activation by phosphorylation of MLKL, the effector of necroptosis [21]. MLKL phosphorylation triggers conformational changes and formation of plasma membrane pores by MLKL, leading to cell death [22, 23]. While their roles in necroptosis is clear, new roles are being investigated for both proteins [24–27].

Apoptosis is the only RCD pathway known to be activated by UVB in dermal fibroblasts and in epidermal keratinocytes [28–31]. We have previously shown that even after apoptosis inhibition, no other RCD (namely ferroptosis, necroptosis and parthanatos) were triggered by UVB, making apoptosis the only measurable cell death induced in normal human dermal fibroblasts (NHDF) [29]. However, previous data showed that RIPK3 transcripts is increased in NHDF treated by chronic UVB doses [32], making RIPK3 a protein of interest in the study of cell response to UVB. RIPK3, through MLKL-mediated necroptosis, was found to be involved in UVA-induced retina and cornea cell death [33, 34]. Those results led us to investigate the involvement of RIPK3 and MLKL in UVB-induced cell death in NHDF.

In this project, we have used siRNA directed against RIPK3 and MLKL to evaluate their role in UVB-induced cell death. As both proteins are known actors of necroptosis, we also investigated a potential UVB-induced necroptosis in NHDF. Our results show that RIPK3 and MLKL both play opposite role in UVB-induced cell death, independently of necroptotic pathway. RIPK3 protect NHDF from UVB-induced cell death, while MLKL sensitises irradiated cells to cell death. We also showed that RIPK3 does not affect caspases activation. On the contrary, MLKL is directly involved in UVB-induced apoptosis. RIPK3 and MLKL are thus two new actors of UVB-induced cell death in NHDF.

## Results

### RIPK3 protects NHDF from UVB-induced cell death

**Fig. 1:**
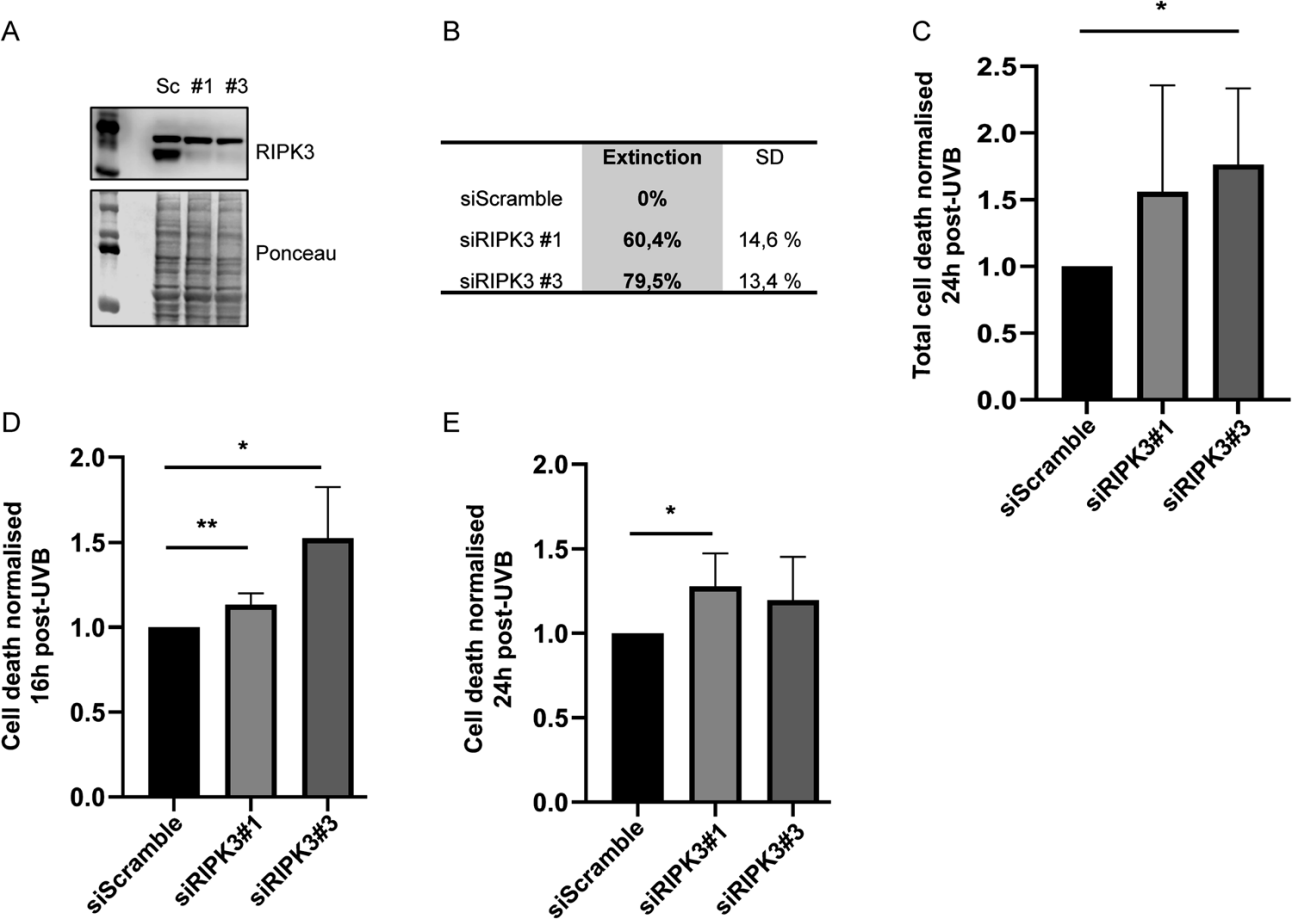
UVB-induced cell death is increased by RIPK3 depletion. NHDF were transfected with siRIPK3 #1 or #3, or by a control siRNA (siScramble; Sc). SiRNA-mediated protein knockdown of RIPK3 was evaluated by Western Blot using RIPK3 antibody (**A**) and quantified (**B**). Following UVB irradiation, UVB-induced cell death was determined by flow cytometry 24 h post-exposition in transfected NHDF (**C**). Cell death was also evaluated by CellTOX assay 16 h (**D**) and 24 h (**E**) post-UVB. *N* = 4, **p-value < 0.01, *p-value < 0.05, t-test. RIPK3 KD leads to an increase in UVB-induced cell death at 16 and 24 h post-UVB, indicating that RIPK3 protects UVB-exposed NHDF from cell death.

We have previously demonstrated that apoptosis is the main pathway in NHDF exposed to UVB, while necroptosis, ferroptosis and parthanatos are not activated [29]. Nevertheless, we have also shown that transcription of RIPK3 was increased after chronic UVB doses in NHDF [32]. We thus investigated the role of RIPK3 in UVB-induced cell death by using siRNA-mediated knock-down (KD). Transfection of RIPK3 siRNA (siRIPK3) #1 and #3, led to 60 and 79% RIPK3 extinction in NHDF, respectively (Fig. 1A, B). UVB-induced cell death was evaluated using two assays, i.e. staining of Annexin V and propidium iodide (PI) measured by flow cytometry and a CellTOX assay. NHDF were 1.5 and 1.7 times more sensitive to UVB-induced cell death measured by flow cytometry at 24 h post-irradiation, when cells were transfected with siRIPK3 #1 and #3, respectively (Fig. 1C). CellTOX assays confirmed those results. Indeed, 16 h post-irradiation, cell death was significantly increased in cells transfected by siRIPK3 #1 and #3 (Fig. 1D). At 24 h post-UVB, cell death measured by CellTOX was still increased by a factor of 1.2 compared to siScramble control (Fig. 1E), but this was significant only with siRIPK3 #1. Those results indicated that RIPK3 KD increases UVB-induced cell death, showing that RIPK3 protects NHDF from UVB-induced cell death.

### MLKL sensitizes NHDF to UVB-induced cell death

**Fig. 2:**
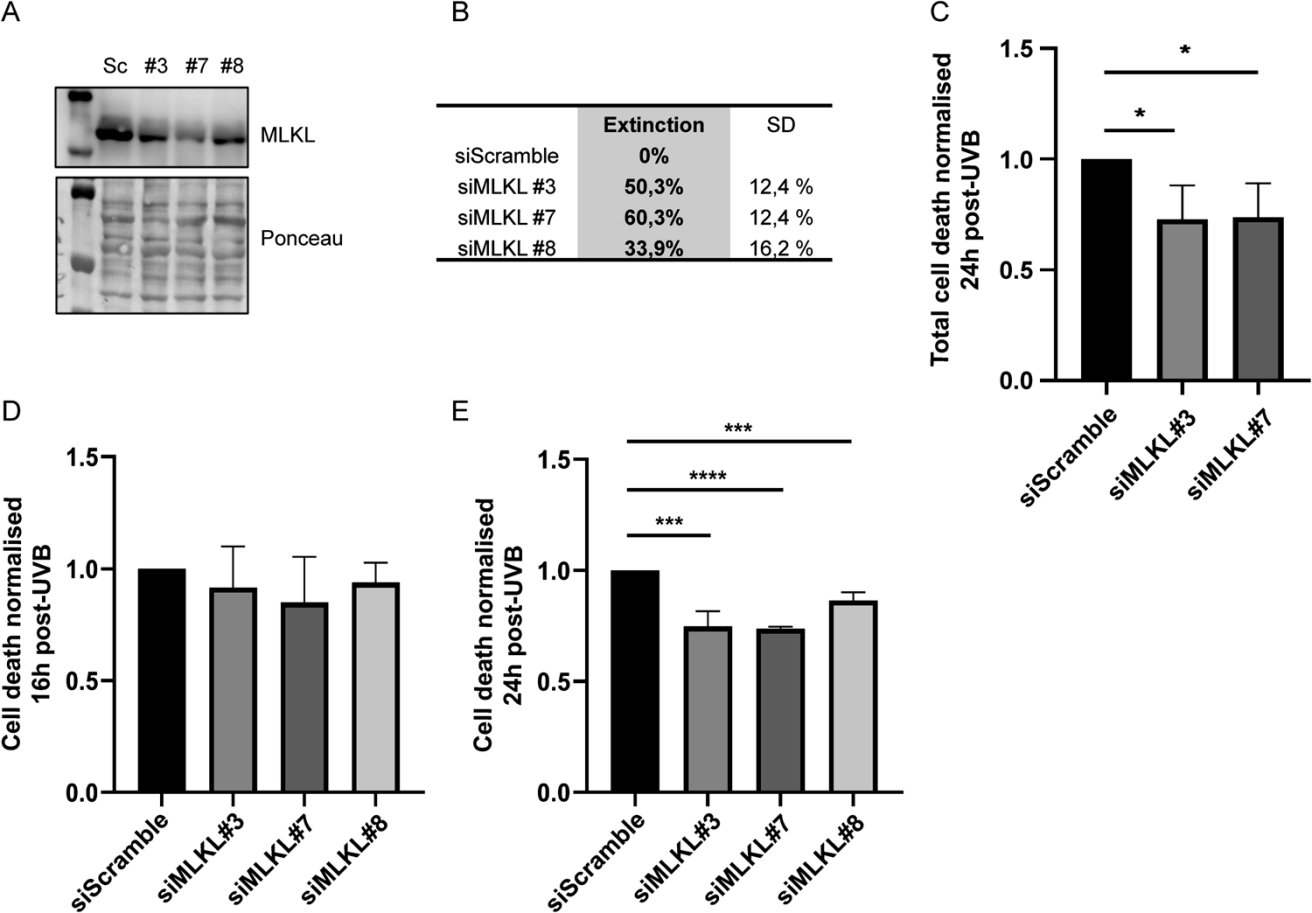
UVB-induced cell death is partially prevented by MLKL depletion. NHDF were transfected by siMLKL #3, #7 or #8, or by a control siRNA (siScramble; Sc). SiRNA mediated protein knockdown of MLKL was evaluated by Western Blot (**A**) and quantified (**B**). UVB-induced cell death was evaluated by flow cytometry 24 h post-exposition in transfected NHDF (**C**). Cell death was also evaluated by CellTOX assay 16 h (**D**) and 24 h (**E**) post-UVB. *N* = 4, ****p < 0,001, ***p < 0,005, *p-value < 0.05, t-test. MLKL KD leads to a decrease in UVB-induced cell death at 24 h post-UVB, indicating that MLKL sensitises NHDF to UVB-induced cell death.

Since RIPK3 was found to be involved in NHDF survival post-UVB, MLKL role in UVB-induced cell death was also investigated by using siRNA-mediated KD. Transfection of NHDF by siMLKL #3, #7 and #8 led to a 50, 60, and 33% MLKL extinction, respectively (Fig. 2A, B). Following a lethal UVB exposure, NHDF cell death was measured by flow cytometry and CellTOX assays. SiMLKL transfection led to a significant decrease in NHDF cell death 24 h post-irradiation, measured by the two assays (Fig. 2C, E). Same, but non-significant, tendency was observed at 16 h post-UVB by CellTOX (Fig. 2D). Indeed, 24 h post-UVB, siMLKL #3 and #7 reduced cell death by a factor of 1.3 compared to siScramble in the two tests (Fig. 2C, E), while siMLKL #8 decreased cell death measured by CellTOX by a factor of 1.15 (Fig. 2E).

### RIPK3 and MLKL role in UVB-induced cell death is independent of necroptosis

**Fig. 3:**
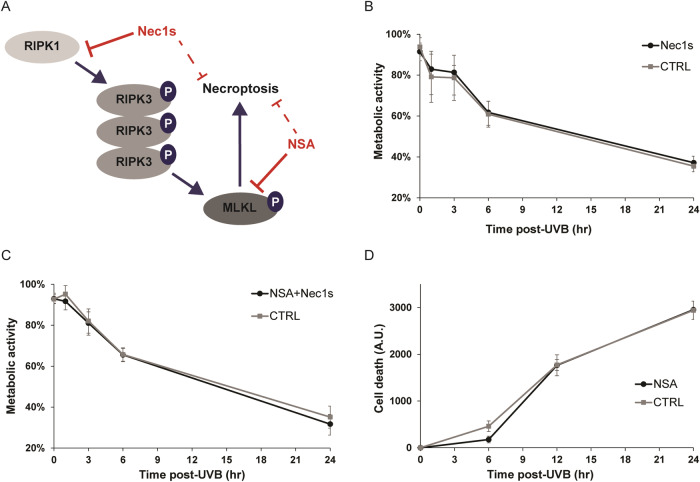
Necroptosis is not induced by UVB in NHDF. NHDF were treated by necroptotic inhibitors Necrosulfonamide (NSA, 2 µM), Necrostatin-1s (Nec1s, 100 µM) or by a combination of both (NSA+Nec1s) and irradiated by a lethal UVB dose. RIPK1, RIPK3 and MLKL implication in necroptosis is schematised in (**A**) with Nec1s et NSA actions. Nec1s inhibits RIPK1 activation while NSA blocks MLKL conformational changes, inhibiting necroptosis. Viability was assessed using MTS assay post-UVB, after Nec1s treatment (**B**) and the combination of NSA+Nec1s (**C**). Cell death was measured post-UVB after NSA treatment by CellTOX assay (**D**). *N* = 4, multiple t-test. Neither inhibitors could prevent UVB-induced viability loss and cell death.

RIPK3 and MLKL are involved in UVB-induced cell death (Figs. 1, 2), but with opposite effects. Since RIPK3 and MLKL are known proteins of the necroptotic pathway [16], we determined their implication in the activation of necroptosis in NHDF. First, necroptosis inhibitors were used to evaluated necroptosis activation following a lethal UVB dose. Necrosulfonamide (NSA), which inhibits MLKL necroptotic activity [35, 36], and Necrostatin-1s (Nec1s), which blocks RIPK1-induced necrosome formation [37] were used (Fig. 3A). After necroptosis inhibition, metabolic activity and cell death was assessed post-UVB. As expected, a lethal UVB exposure induced a decrease in metabolic activity (Fig. 3B, C) and a concomitant increase in cell death (Fig. 3D). Nec1s or NSA alone or both combined had no effect on metabolic activity reduction and cell death induced by UVB (Fig. 3B–D). Those results show that necroptosis is not activated by UVB in NHDF.

**Fig. 4:**
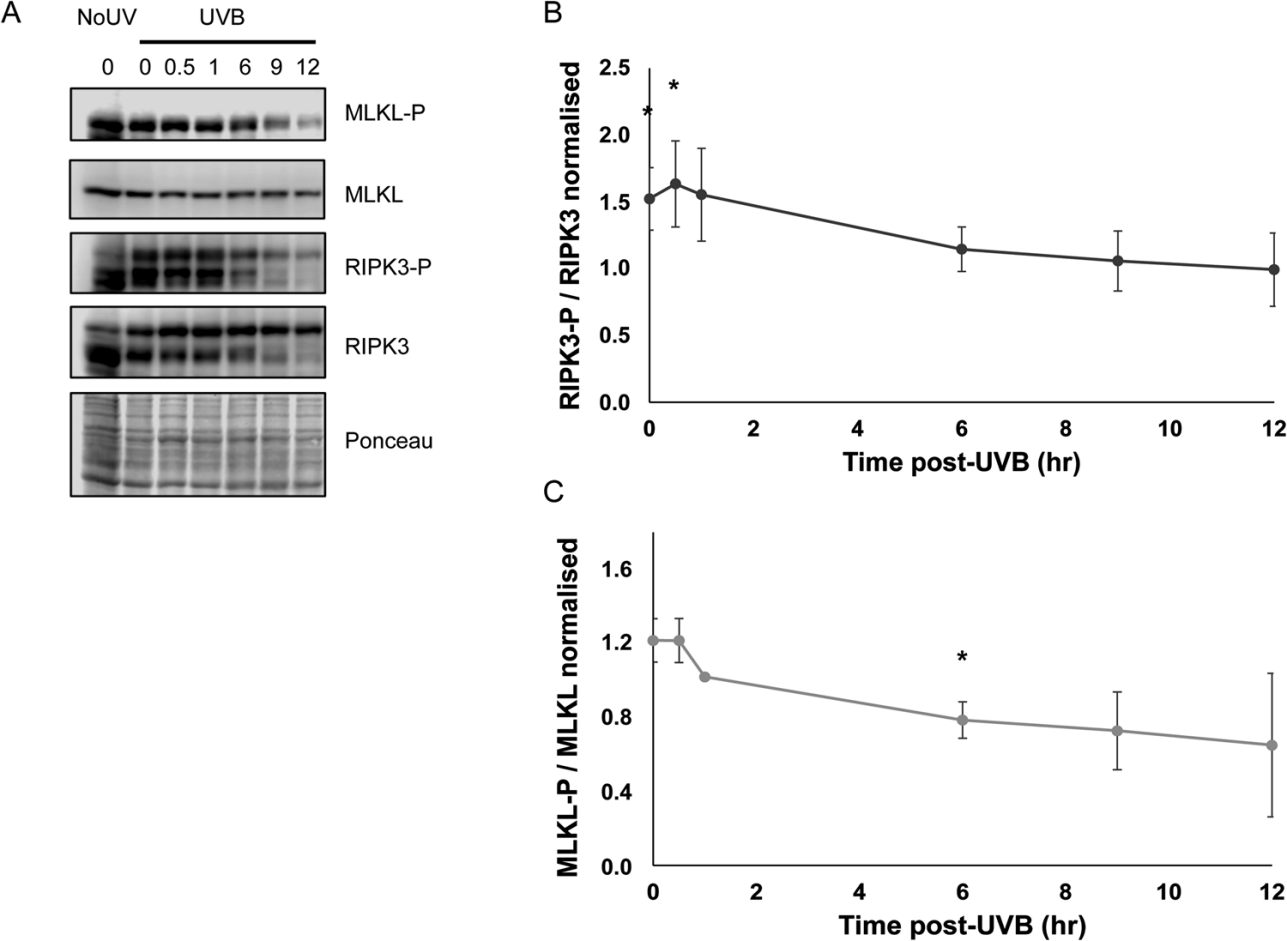
RIPK3 and MLKL necroptotic phosphorylation changes. NHDF were irradiated by a lethal UVB dose (UVB) or not (NoUV). At 0, 0.5, 1, 6, 9, and 12 h post-UVB, levels of phosphorylated RIPK3 (RIPK3-P), RIPK3, phosphorylated MLKL (MLKL-P) and MLKL were assessed by Western Blot (**A**). Ponceau staining was used as loading control. Quantification of the ratio RIPK3-P / RIPK3 in irradiated cells is presented in (**B**). The ratio RIPK3-P / RIPK3 in the NoUV condition was considered as the baseline (value of 1). Quantification of the ratio MLKL-P / MLKL in irradiated cells is presented in (**C**); a value of 1 was attributed to the ratio MLKL-P / MLKL of the NoUV condition. *N* = 3, *p-value < 0.05, multiple t-test. The proportion of RIPK3-P increased between 0 and 0.5 h post-UVB and then reached the NoUV level, while proportion of MLKL-P decreases significantly at 6 h post-UVB compared to the NoUV level.

Phosphorylation of RIPK3 (RIPK3-P) and MLKL (MLKL-P), involved in necroptosis [38], were also analysed following UVB irradiation. The proportion of RIPK3-P / RIPK3 and MLKL-P / MLKL in UVB-exposed NHDF was compared to an unirradiated control (NoUV) (Fig. 4A). The analysis showed that RIPK3-P / RIPK3 increases quickly post-UVB (between 0 and 0.5 h post-UV), but then decreases to reach the NoUV level (Fig. 4B). On the contrary, MLKL-P / MLKL in irradiated NHDF decreases significantly below the NoUV level, placed at 1, at 6 h post-UVB (Fig. 4C). Thus, RIPK3 and MLKL necroptotic-specific phosphorylations are unlikely to be involved in UVB-induced cell death.

### RIPK3 does not affect apoptosis, while MLKL sensitise NHDF to UVB-induced apoptosis

**Fig. 5:**
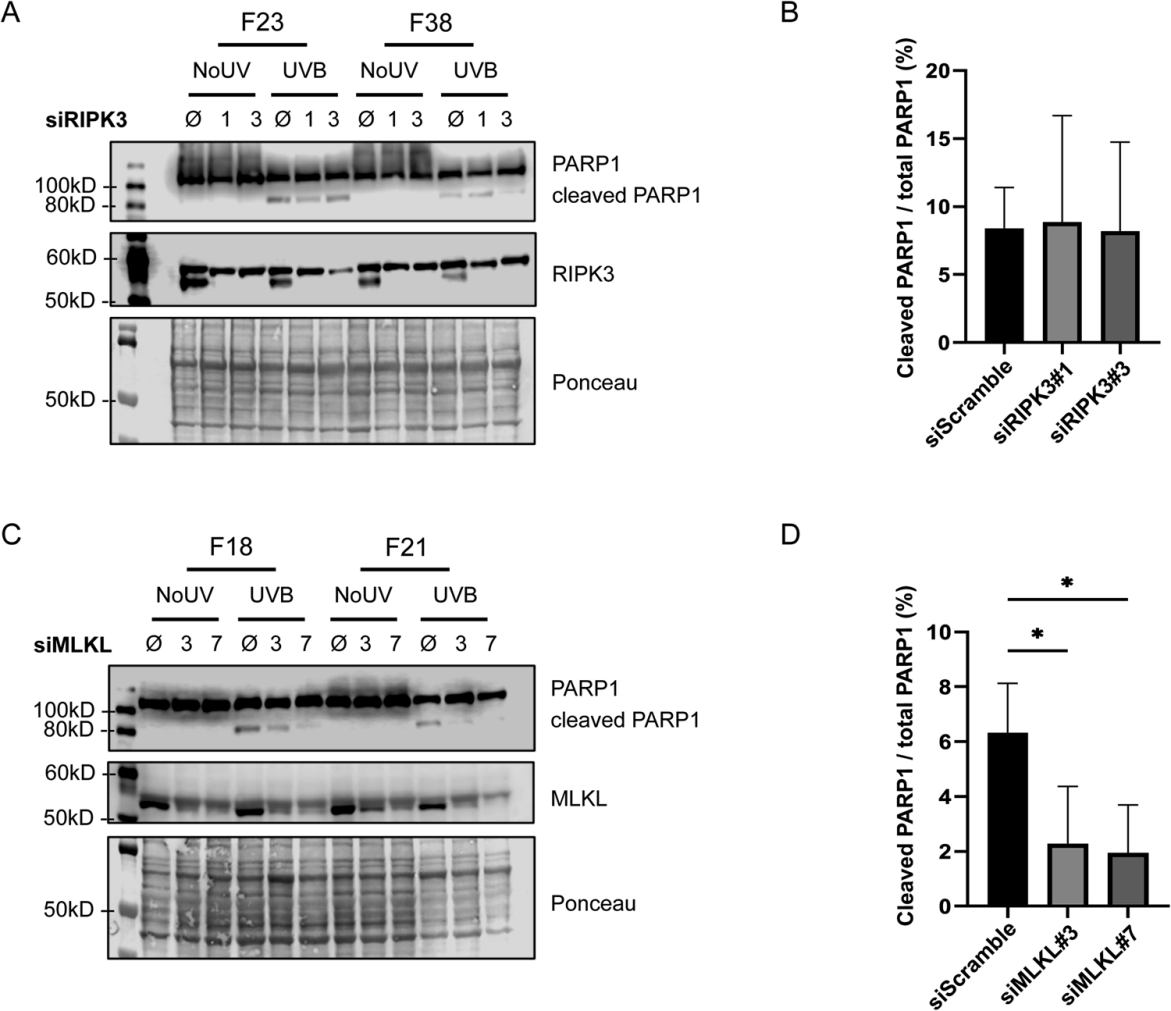
RIPK3 and MLKL involvement in UVB-induced apoptosis. NHDF were transfected by siRIPK3 #1 and #3 in (**A**, **B**), or by siMLKL #3 and #7 in (**C**, **D**). siScramble (Ø) was used as a control. The levels of PARP1 and its cleaved product, RIPK3 and MLKL were determined by Western Blot. Ponceau staining was used as loading control. Quantification of the ratio cleaved PARP1 / total PARP1 is depicted in (**B**) for siRIPK3 and in (**D**) for siMLKL treated cells. Total PARP1 is determined as the sum of cleaved PARP1 (89 kD) and PARP1 (116 kD). *N* = 4, *p-value < 0.05, *t*-test. RIPK3 KD did not change the proportion of cleaved PARP1, while MLKL KD decreased cleaved PARP1 / total PARP1, indicating that MLKL plays a role in UVB-induced apoptosis.

As we showed that RIPK3 and MLKL roles in UVB-induced cell death were independent of necroptosis (Figs. 3 and 4), we examined their involvement in UVB-induced apoptosis. Using siRNA directed against RIPK3 or MLKL, we measured the cleavage of PARP1, a known target of caspases [39] (Fig. 5). As expected, we observed PARP1 cleavage post-UVB in NHDF, reflecting caspases activation and apoptosis (Fig. 5A, C). NHDF transfected by siRIPK3 had the same level of cleaved PARP1 / total PARP1 than siScramble control cells (Fig. 5B), indicating that RIPK3 role in survival post-UVB is independent of apoptosis. On the contrary, cells transfected by siMLKL had a lower proportion of cleaved PARP1 (Fig. 5D), which means that MLKL KD led to a decrease in caspases activity. Those results, confirmed by caspase-3 activation measurement (Figure S2), point out a direct role for MLKL in UVB-induced apoptosis.

## Discussion

UVB is recognized as a complete carcinogen [12, 40]. Cell death is a mechanism known to supress damaged cells and thus act as a cancer prevention mechanism [15, 41]. We have previously demonstrated that only apoptosis is measurable in NHDF exposed to a lethal UVB dose [29]. Here, we have shed light on the implication of two new actors, RIPK3 and MLKL, on UVB-induced cell death pathway. RIPK3 was found to protect NHDF from UVB cell death, independently of necroptosis and apoptosis. On the other hand, MLKL was found to be directly involved in UVB-induced apoptosis, independently of necroptosis.

RIPK3 and MLKL are known actors of necroptosis, with MLKL being the effector of this cell death pathway [42]. Nevertheless, we have shown that RIPK3 suppression led to an increase in NHDF cell death post-UVB, indicating an unexpected role for RIPK3 in survival. Additionally, since our results confirmed that necroptosis is not activated by UVB, RIPK3 involvement in UVB cell death is independent of necroptosis. Besides, RIPK3 have no direct effect on UVB-induced apoptosis as evidenced by the absence of modification in the cleavage of PARP1 post-UVB following RIPK3 siRNA. This suggests a new role for RIPK3 in cell survival. Recently, new roles for RIPK3 have been identified, one of which is in inflammatory response and cytokines production [25, 43], and another one is in promoting kidney fibrosis by an NFκB-AKT pathway [24]. Here we uncover a new role for RIPK3 in survival of UVB-irradiated NHDF, but the mechanism of action for RIPK3 still need to be identified. We verified that RIPK3 suppression was not influencing cell cycle, which could explain, at least in part, the cell survival improvement following UVB exposure (Figure S1A). SiRIPK3 led to small changes in cell cycle distribution, with siRIPK3 #1 leading to a small increase in G2/M phase cells while siRIPK3 #3 led to a decrease in G2/M phase compared to siScramble control cells. RIPK3 is thus unlikely to be involved in cell cycle regulation. Of note, more than 80% of NHDF were supposedly in G1 phase the day of irradiation (Figure S1A). This led us to state two hypotheses, i.e. RIPK3 is involved in DNA damage repair pathway, or RIPK3 activate survival pathway, either by the NFκB-AKT axis [24] or by interacting with RIPK1 via their RHIM domain. RIPK3 implication in cell survival post-UVB will need further investigation.

We uncovered a role of MLKL in UVB-induced apoptosis. Indeed, MLKL suppression by siRNA led to a decrease in cleaved PARP1. We also confirmed that MLKL suppression do not influence cell cycle (Figure S1B). In fact, MLKL involvement in apoptosis is sparsely documented. W. Cao et al showed the involvement of MLKL in apoptosis induced by the Chelerythrine drug. In their work, formation of reactive oxygen species (ROS) triggered MLKL and PERK- eIF2α mutual regulation, leading to apoptosis [26]. ROS can be formed following UV, leading to cellular damage [44–46]. Nevertheless, in a previous study we examined metabolic activity changes post-UVB in NHDF treated with antioxidant, and found no modifications in cell viability [29]. While a link between UVB-induced ROS formation and MLKL role in apoptosis is possible, it seemed unlikely. The relationship between MLKL and caspases activation by UVB should be further investigated to unravel the mechanism of UVB-induced apoptosis.

Here we present new roles for RIPK3 and MLKL, in survival and apoptosis, respectively. We have identified those two proteins as actors of UVB-induced cell death in human dermal fibroblasts. Altogether, this study provides new insight of the apoptotic pathway activated by UVB in skin.

## Materials and Methods

All experiments in this study were performed in accordance with the Declaration of Helsinki, and the research protocol received approval by the CHU de Québec-Université Laval (Québec) institutional ethics committees for the protection of human subjects with written informed patient consent for study participation.

### Cell culture

Normal human diploid fibroblasts (NHDF) were taken from skin biopsies (mastectomy) of 4 healthy women from 18 to 38 years old (F18, F21, F23, F38). Fibroblasts were cultured in Dulbecco’s modified Eagle’s Medium (DMEM; Wisent, Saint-Jean-Baptiste, Canada) supplemented with 5% FBS (Sigma, Oakville, Canada) and 1% penicillin/streptomycin (Wisent) at 37 °C, 5% CO_2_.

### siRNA transfection

RIPK3 siARN (siRIPK3) and MLKL siRNA (siMLKL) used were: siRIPK3 #1 (cat no SI00092792, target sequence ACCGCTCGTTAACATATACAA, Qiagen, Germantown, MD, USA), siRIPK3 #3 (cat no SI00092806, target sequence CAGCCTGATGTCGTGCGTCAA, Qiagen), siMLKL #3 (cat no SI03112249, target sequence TAGGTTTCAAGAGGCCTTATA, Qiagen), siMLKL #7 (cat no SI04903857, target sequence CTCGCTGTTACTTCAGGTTGA, Qiagen) and siMLKL #8 (cat no SI04961390, target sequence ACTGAGACGATTAGAAATCAA, Qiagen). siScramble (AllStars Negative Control siRNA, cat no 1027281, Qiagen) was used as control.

A reverse transfection was performed in NHDF transfected by siMLKL (day 1). A reverse transfection (day 1) followed the next day by a forward transfection (day 2) was performed in NHDF transfected by siRIPK3. At day 4 for siMLKL and day 6 for siRIPK3 conditions, cells were irradiated with a lethal UVB dose of 10,000 J/m^2^ (UVB) or not (NoUV) and then analysed by CellTOX, flow cytometry or Western Blot.

Transfection was performed by mixing siRNA (12 nM final concentration) to opti-MEM (opti-MEM I reduced serum media, Thermofisher Scientific, Burlington, CA) and lipofectamin (Lipofectamin RNAiMAX, Thermofisher Scientific) following manufacturer protocol.

### Necroptosis inhibition

NHDF were treated by necroptosis inhibitors following the protocol previously described [29]. Briefly, confluent NHDF were incubated 30 min with either necroptosis inhibitors or control media at the given concentration in DMEM. Following incubation, NHDF were irradiated with a lethal UVB dose of 30,000 J/m^2^ or not. After irradiation, cells were returned to the incubator in DMEM containing or not the inhibitors until analysed by CellTOX or MTS.

Inhibitors used to block necroptosis were Necrosulfonamide (NSA; R&D System, Bristol, UK) at 2 µM final concentration and Necrostatin-1s (Nec1s; Millipore, Billerica, MA, USA) at 100 µM final concentration. A combination of NSA and Nec1s was used. The control media was a Dimethyl Sulfoxide (DMSO; Sigma) dilution in DMEM.

### UVB irradiation

The UVB source consisted of RPR-3000 lamps with an emission peak at 300 nm (Southern New England Ultraviolet Co., Branford, CT, USA) filtered through a sheet of cellulose acetate to eliminate wavelengths under 295 nm (Kodacel TA-407 clear 0.015 inches; Eastman-Kodak Co., Rochester, NY, USA). Irradiance of this source is 19 W/m^2^ as measured using an UVX radiometer equipped with an UVX-31 sensor (UVP, Upland, CA, USA). NHDF transfected by siRNA and NHDF treated with necroptosis inhibitors were irradiated with a lethal dose of 10,000 J/m^2^ and 30,000 J/m^2^ of UVB, respectively. A lethal dose of 20,000 J/m^2^ was used to examine RIPK3 and MLKL phosphorylation post-UVB by Western Blot.

### Metabolic activity assay - MTS assay

As previously described [29], MTS assay was performed (CellTiter 96® Aqueous non-radioactive Cell proliferation assay, Promega, Madison, WI, USA) to assess metabolic activity. Briefly, cells were incubated at 37 °C for 0 to 24 h post-UVB. For each time-point, MTS reagents were added on cells and incubated 30 min to 1 h. Absorbance at 490 nm was then measured. Irradiated cells (UVB) were normalised on un-irradiated cells (NoUV) of the same condition, then NHDF treated with necroptosis inhibitors were compared to cells treated with control media.

### Cell death assay - cellTOX

Following UVB exposure, a cell death assay was performed (CellTox™ Green Cytotoxicity Assay; Promega) according to the manufacturer protocol. Briefly, following UVB irradiation, the fluorescent dye was added directly in the culture media. Fluorescence, representing cell death, (excitation peak at 480 nm, emission peak at 530 nm) was then measured at different time-point post-UVB, i.e., 0 h, 6 h, 12 h, 24 h in necroptosis inhibited cells, and 0 h, 16 h, 24 h in siRNA transfected cells. Value from un-irradiated cells (NoUV) were subtracted to fluorescence from irradiated cells to remove the background. Fluorescence of each condition was relative to the 0 h post irradiation.

For siRNA assay, siRIPK3 or siMLKL conditions were normalised on siScramble condition for each culture.

### Cell death assay - flow cytometry

After irradiation, NHDF were harvested at 24 h post-UVB. Cells were then labelled with propidium iodide (PI) and Annexin V-FITC (AV) to analyse cell death by flow cytometry, using the kit FITC Annexin V Apoptosis Detection Kit I (cat no 556547, BD Biosciences, San Diego, CA, USA). Total cell death was measured by adding the percentage of PI + / Annexin V– cells, PI- / Annexin V+ cells and double positive cells. Value from un-irradiated cells (NoUV) were subtracted to fluorescence from irradiated cells to remove the background. siRIPK3 or siMLKL conditions were normalised on siScramble condition for each culture.

### Western blot analysis

NHDF transfected by siRNA were harvested the day of theoretical irradiation to examine RIPK3 and MLKL knock-down. SiRNA transfected NHDF were irradiated or not (NoUV) and then harvested 24 h post-UVB to study PARP1 cleavage. After NHDF irradiation and necroptosis inhibition, cells were harvested 0 to 12 h post-UVB to study RIPK3 and MLKL phosphorylation, a NoUV control was harvested at 0 h post-UVB. NHDF lysis was performed in RIPA buffer (1% NP 40, 0.5% Sodium Deoxycholate, 0.1% SDS, 150 mM NaCl, 50 mM Tris HCl pH8) complemented with cOmplete^™^, EDTA-free Protease Inhibitor Cocktail (Roche, Mannheim, Germany) and phosphatase inhibitor PhosSTOP^™^ (Roche). Total proteins were quantified using BCA quantification (Pierce BCA Protein Assay Kit, ThermoFisher Scientific). Proteins were run on SDS-PAGE and visualised by SuperSignal™ West Pico PLUS Chemiluminescent Substrate (Thermo Fisher Scientific). Membranes were scanned using the C-DiGit Blot Scanner (LI-COR Biosciences, USA) and analysed with Image Studio Lite software (LI-COR Biosciences). Each membrane was stained with Ponceau to have protein level control.

Antibodies α-MLKL (3H1, #MABC604, 1:1000, Millipore, Oakville, CA), α-MLKL phospho ser358 (#91689, 1:500, Cell Signaling Technology, Danvers, MA, USA), α-PARP (F2, #sc-8007, 200 ug/mL, 1:500, Santa Cruz Biotechnology, Dallas, TX, USA), α-RIPK3 (B2, Sc-374639, 1:1000, Santa Cruz Biotechnology) or α-RIPK3 (6E6.2, #MABC28, 1:1000, Millipore, Oakville, CA) and α-RIPK3 phospho ser227 (#Ab209384, 0,731 mg/mL, 1:500, Abcam, Cambridge, UK) were used.

### Statistical analysis

Data are presented with mean +/− SD, statistical significance was evaluated using GraphPad Prism8 software (GraphPad Software Inc., San Diego, CA, USA). The “N” indicates the number of human primary cultures used. A *p*-value < 0.05 was considered statistically significant.

Unpaired t-tests were used to compare individually each siRNA (siMLKL or siRIPK3) to the siScramble control. Multiple unpaired *t*-test were used to compare two conditions at each timepoint, i.e., inhibitor condition compared to control media or UVB compared to NoUV condition. Hence, Western Blot results of phosphorylated MLKL (or RIPK3) / total MLKL (or RIPK3) were analysed by comparing each timepoint post-UVB to the NoUV control placed at 1.

### Supplementary information

Supplementary material

Uncropped Western Blots

## Data Availability

No datasets were generated or analyzed during the current study.
