# Supplementary material for: MLKL, a new actor of UVB-induced apoptosis in human diploid dermal fibroblasts
Source: Cell Death Discov. 2024 May 14;10:232. doi: 10.1038/s41420-024-02004-4 (PMC11093999; doi:10.1038/s41420-024-02004-4)
Supplement: Supplementary file 1 — Supplementary material [file 41420_2024_2004_MOESM1_ESM.docx]

**Supplementary material**

***MLKL, a new actor of UVB-induced apoptosis in human diploid dermal fibroblasts***

Anne-Sophie Gary^1,2^ , Sophie Amouret^1,2^, Alicia Montoni^1,2^ and Patrick J. Rochette^1,2, 3,^*

1. Centre de Recherche du CHU de Québec – Université Laval, Axe Médecine Régénératrice, Hôpital du Saint-Sacrement, Québec, Qc, Canada

2. Centre de Recherche en Organogénèse Expérimentale de l'Université Laval/LOEX, Université Laval, Québec, Qc, Canada.

3. Département d’Ophtalmologie et ORL - chirurgie cervico-faciale, Université Laval, Québec, Qc, Canada.

* To whom correspondence should be addressed. Tel: (418) 682-7568; Fax: (418) 682-8000;
E-mail : Patrick-J.Rochette@crchudequebec.ulaval.ca

**Supplemental figure**


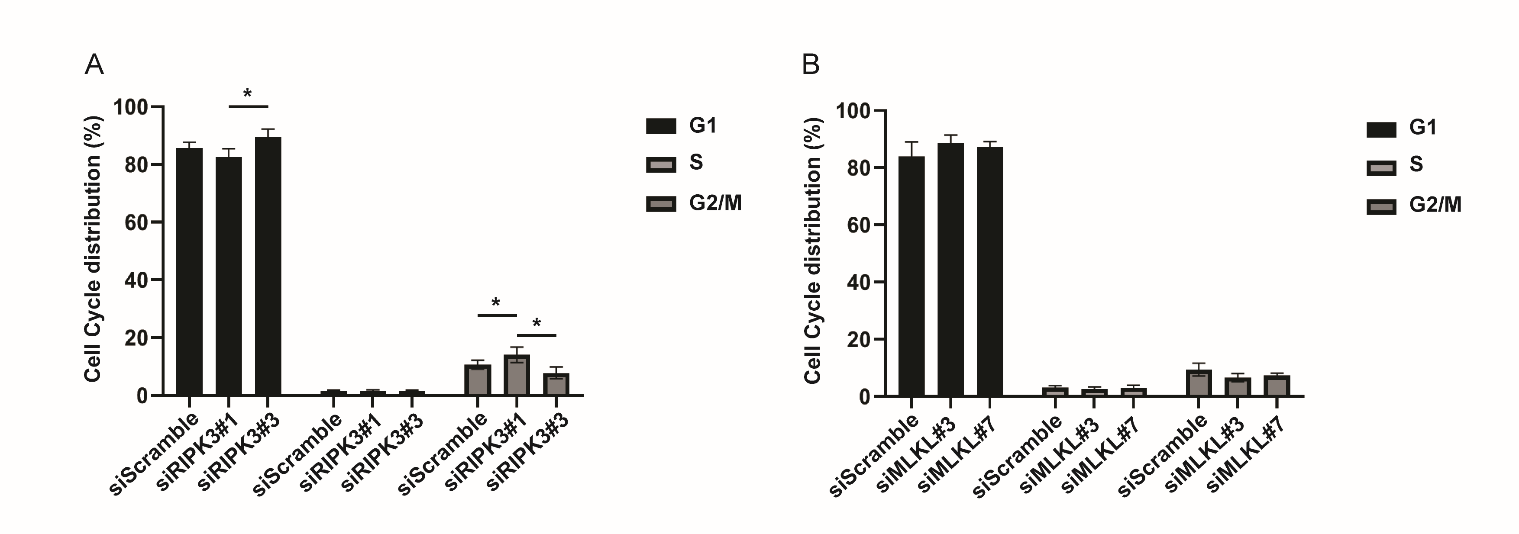


Figure S1: RIPK3 and MLKL deletion impact on cell cycle distribution. NHDF were transfected by siRIPK3 #1 and #3 in (A), or by siMLKL #3 and #7 in (B). Each time, siScramble was used as a control. At the day of theoretical irradiation, cells were harvested, fixed in 70% ethanol for 30 min on ice, washed in PBS and then incubated 30 min with propidium iodide (PI, 50 µg/ml) at 37ºC. Cell cycle distribution (G1, S and G2/M phase) was assessed by flow cytometry. N=4, * p-value < 0.05, Two-way Anova et post-test Tukey. Small changes are visible in siRIPK3 transfected cells (G2/M, siScramble : 10,7 %; siRIPK3#1 : 14,1 %; siRIPK3#3 : 7,8%). No changes are visible using siMLKL compared to siSramble.


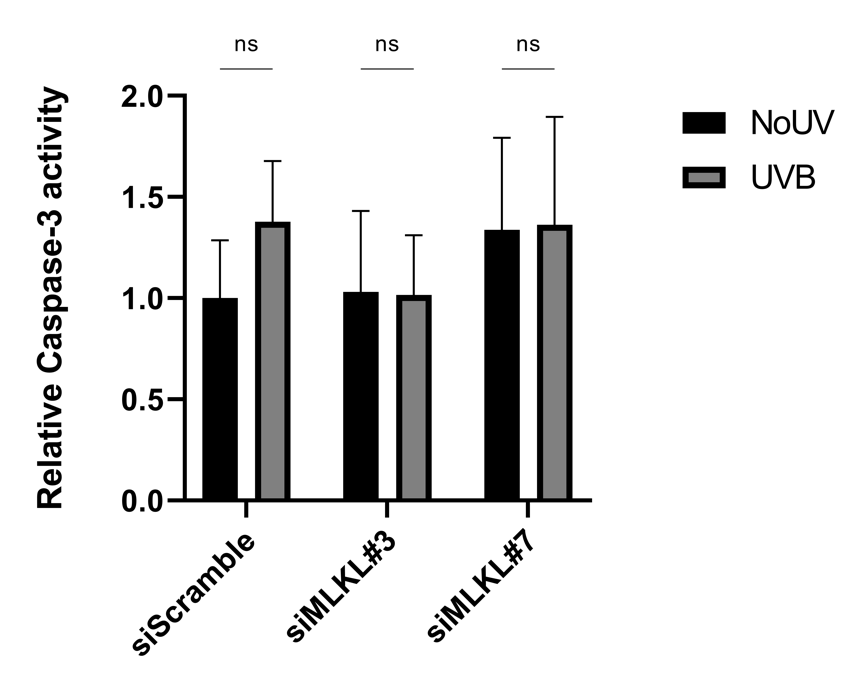

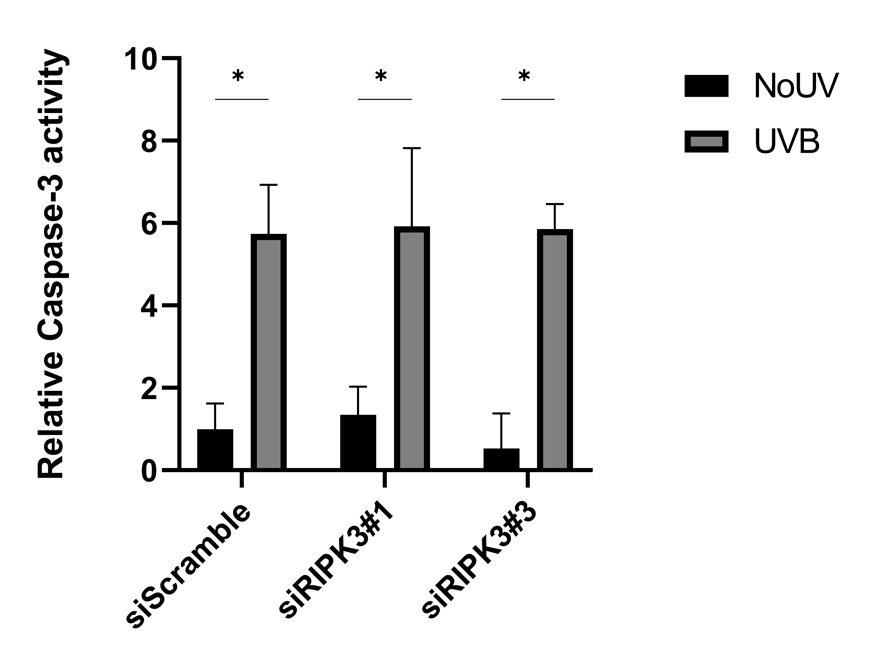


Figure S2: NHDF were transfected by siRIPK3 #1 and #3 (Left), or by siMLKL #3 and #7 (Right). siScramble was used as a control. Eight hours following UVB irradiation (20 kJ/m^2^) of NHDF, cells have been harvested and the levels of cleaved caspase-3 were determined using ApoAlert Caspase-3 colorimetric assay kit and read on a spectrophotometer at 405 nm. N=3, * p-value < 0.05, t-test. RIPK3 KD did not change the proportion of cleaved caspase-3, while MLKL KD decreased cleaved caspase-3 level. This confirms the results from cleaved PARP1 / total PARP1 (figure 5), indicating that MLKL plays a role in UVB-induced apoptosis.
