# Supplementary material for: MLKL, a new actor of UVB-induced apoptosis in human diploid dermal fibroblasts
Source: Cell Death Discov. 2024 May 14;10:232. doi: 10.1038/s41420-024-02004-4 (PMC11093999; doi:10.1038/s41420-024-02004-4)

Western Blot

Figure 1 :

Western-blot order samples:

L – - Scrbl - #3 - #7 – Scrbl - #3 - #7 – Scrbl - #3 - #7 – Scrbl - #3 - #7

F18 F21 F23 F38

With Ladder = L; SiScramble = Scrbl; siRIPK3#3 = #3; siRIPK3#7 = #7; Fibroblast primary culture name = F18, F21, F23 and F38

siRIPK3

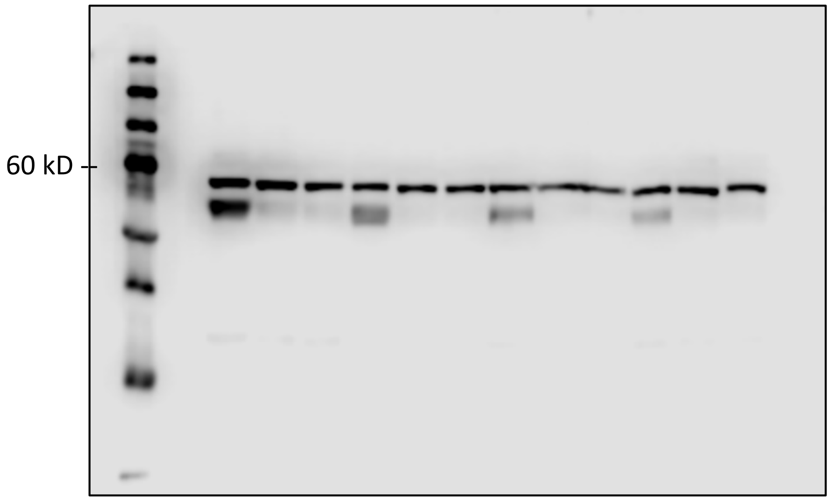

Associated Ponceau

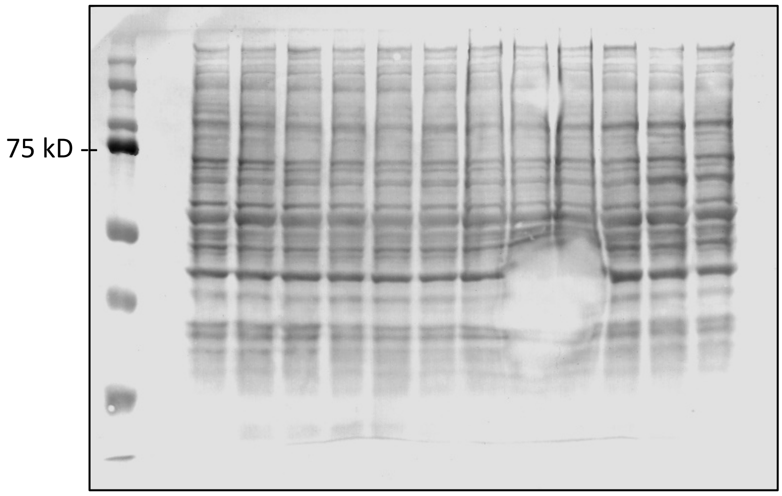

Figure 2 :

Western-blot order samples:

F18 F23

L – Scrbl - #3 - #7 - #8 – NoSi – Scrbl - #3 - #7 - #8 – NoSi - - L

With Ladder = L; SiScramble = Scrbl; siMLKL#3 = #3; siMLKL#7 = #7; siMLKL#8 = #8; Fibroblast primary culture name = F18, F21, F23 and F38

siMLKL

60 kD

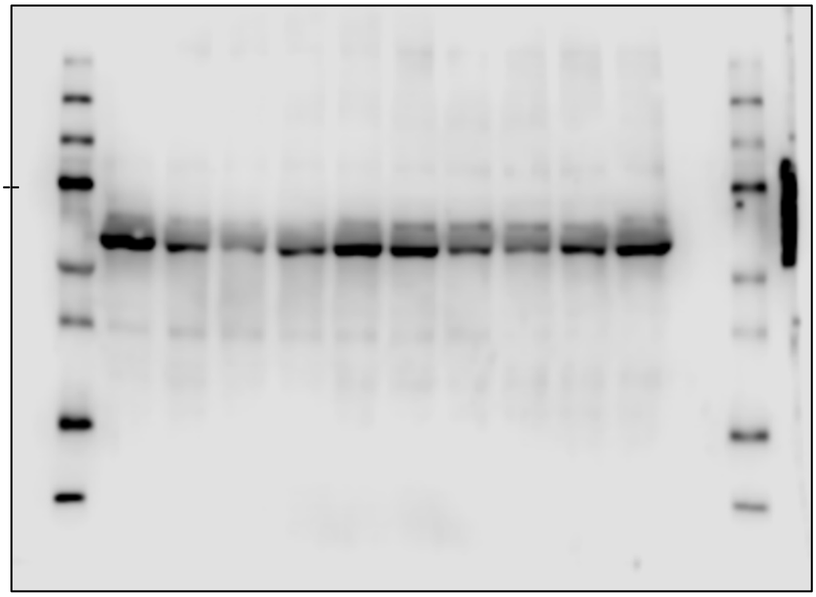

Associated Ponceau

75 kD

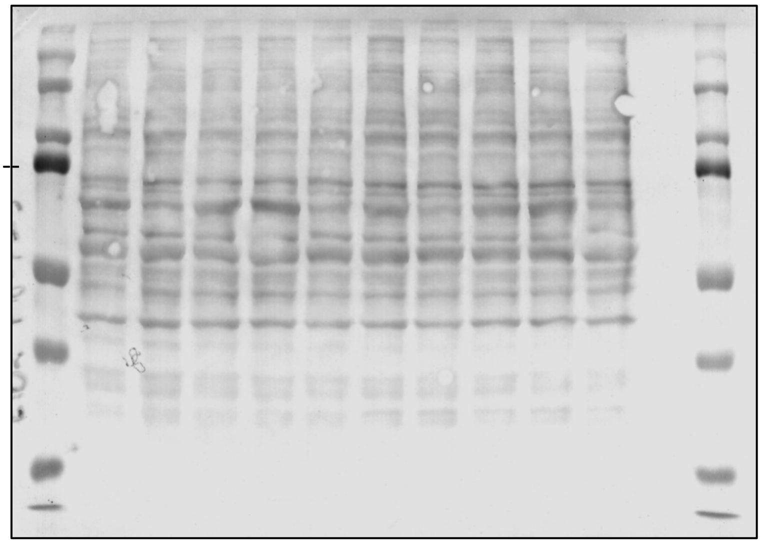

Figure 4

Western-blot order samples:

NoUV NoUV+A CLUV CLUV+A L
0h 0 0.5 1 6 9 12 0h 0 0.5 1 6 9 12 Time post-irradiation (hrs)

With Ladder =L; unirradiated cells (Ctrl) = NoUV; irradiated cells = NoUV+A; chronic low UVB-dose (repeated irradiation) = CLUV; chronic low UVB-dose plus an acute irradiation = CLUV+A.

After Ponceau staining, membrane was cut under 75kD and above 37kD. Between each antibody, membrane was strip. Antibodies were done in this order : Phospho-MLKL phospho-RIPK3, RIPK3 and MLKL

MLKL-P

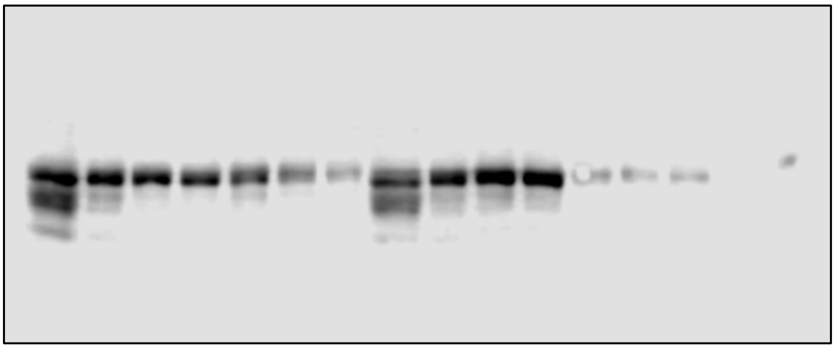

MLKL

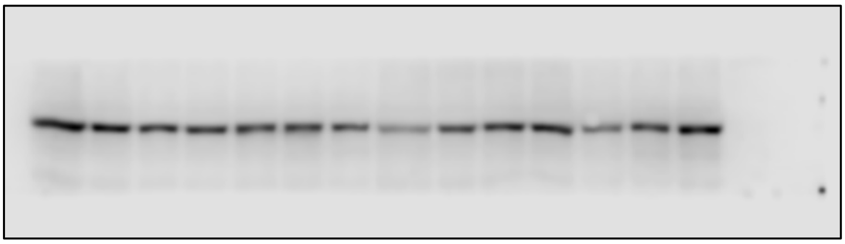

RIPK3-P

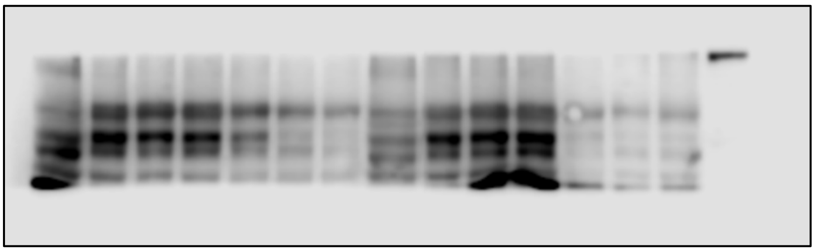

RIPK3

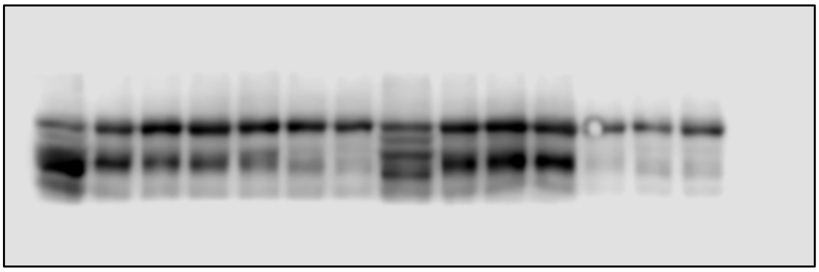

Associated Ponceau

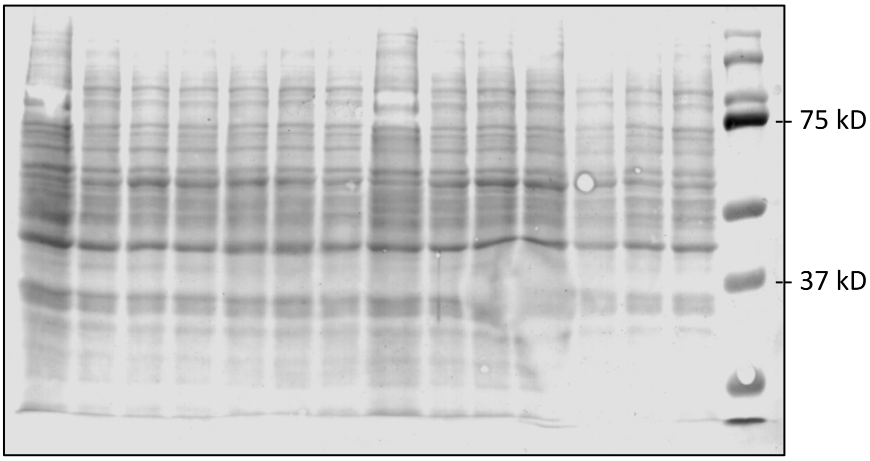

Figure 5 A

Western-blot order samples:

L – - Scrbl - #3 - #7 – Scrbl - #3 - #7 – Scrbl - #3 - #7 – Scrbl - #3 - #7

NoUV UV NoUV UV

F23 F38

With Ladder = L; SiScramble = Scrbl; siRIPK3#3 = #3; siRIPK3#7 = #7; unirradiated cells = noUV; irradiated cells = UV; Fibroblast primary culture name = F23 and F38

Between each antibody, membrane was strip. Antibodies were done in this order: PARP then RIPK3.

PARP

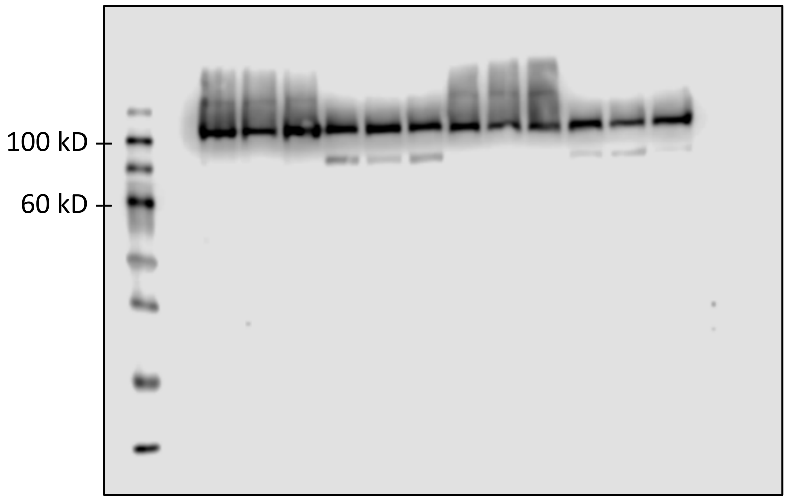

RIPK3

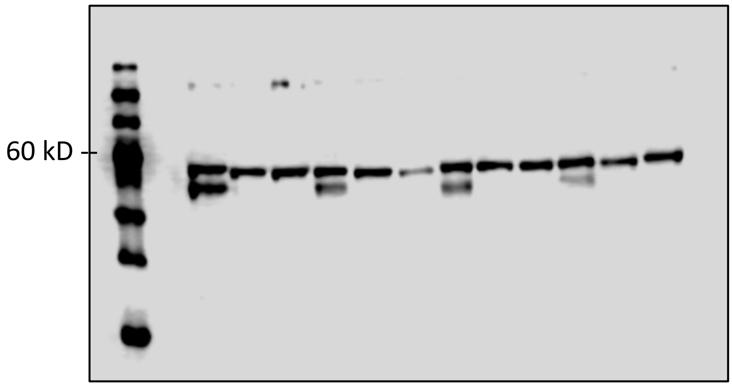

Ponceau

75 kD

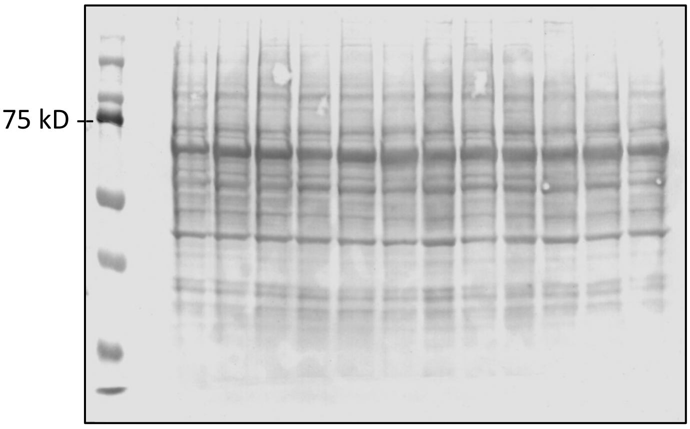

Figure 5 B

Western-blot order samples:

NoUV UV NoUV UV

L – Scrbl - #3 - #7 – Scrbl - #3 - #7 – Scrbl - #3 - #7 – Scrbl - #3 - #7 - - L

F18 F21

With Ladder = L; SiScramble = Scrbl; siMLKL#3 = #3; siMLKL#7 = #7; siMLKL#8 = #8; unirradiated cells = noUV; irradiated cells = UV; Fibroblast primary culture name = F18 and F21

After Ponceau staining, membrane was cut under 75kD and above 37kD for MLKL staining. After stripping the membrane, staining of PARP was done, the cutted-membrane above 75kD was also stained for PARP, the two cutted parts were revealed at the same time.

PARP

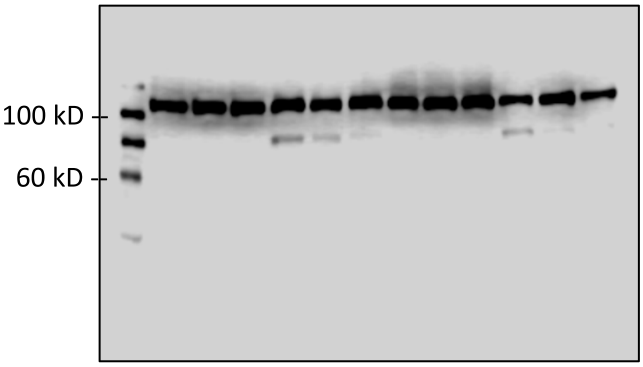

MLKL

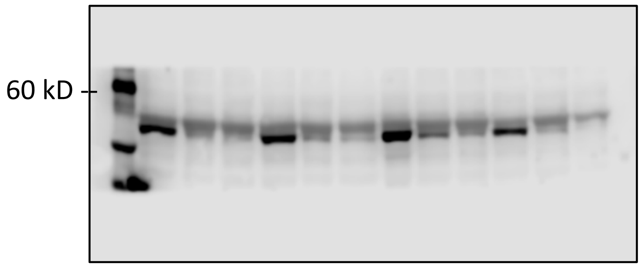

Ponceau

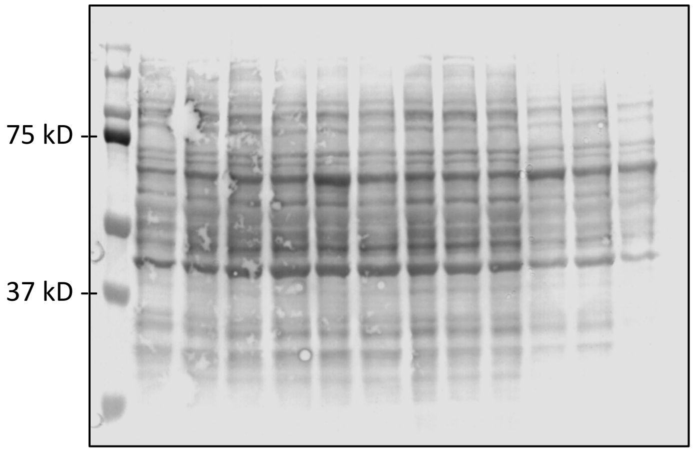

Supplement: Supplementary file 2 — Uncropped Western Blots [file 41420_2024_2004_MOESM2_ESM.pdf]
